# Ornithology: Third Book of Natural History, Prepared for the Use of Schools and Colleges

**Published:** 1842-05-21

**Authors:** 


Ornithology: *Third Book of Natural History, prepared for the use of Schools and Colleges.* By W. S. W. Ruschenberger, M. D., Surgeon in the U. S. Navy, &c. &c. Philadelphia, Turner & Fisher, 1842. 12 mo. pp. 125.

We did not receive the two first volumes of Dr. Ruschenberger's Series of Natural History. The third, the Ornithology, we have great pleasure in recommending as an excellent elementary manual on the subject. This series of "primers" are, in the main, derived from the text of MM. Milne Edwards and Achille Comte, the distinguished professors of Natural History in Paris. With this, however, Dr. Ruschenberger has incorporated other matter. We are happy to learn that they have been very generally introduced as text-books into schools and colleges.

